# Structural and Phase Transformations and Physical and Mechanical Properties of Cu-Al-Ni Shape Memory Alloys Subjected to Severe Plastic Deformation and Annealing

**DOI:** 10.3390/ma14164394

**Published:** 2021-08-05

**Authors:** Alexey E. Svirid, Vladimir G. Pushin, Natalia N. Kuranova, Vladimir V. Makarov, Yuri M. Ustyugov

**Affiliations:** Mikheev Institute of Metal Physics, Ural Branch, Russian Academy of Sciences, Ekaterinburg 620108, Russia; pushin@imp.uran.ru (V.G.P.); kuranova@imp.uran.ru (N.N.K.); makarov@imp.uran.ru (V.V.M.); ustyugov@imp.uran.ru (Y.M.U.)

**Keywords:** copper shape memory alloys (SMAs), severe plastic deformation (SPD), high pressure torsion (HPT), heat treatment (HT), thermoelastic martensitic transformation (TMT), shape memory effect (SME), mechanical properties, ductility

## Abstract

Using the methods of electron microscopy and X-ray analysis in combination with measurements of the electrical resistance and magnetic susceptibility, the authors have obtained data on the peculiar features of pre-martensitic states and martensitic transformations, as well as subsequent decomposition, in the alloys with shape memory effect of Cu–14wt%Al–3wt%Ni and Cu–13.5wt%Al–3.5wt%Ni. For the first time, we established the microstructure, phase composition, mechanical properties, and microhardness of the alloys obtained in the nanocrystalline state as a result of severe plastic deformation under high pressure torsion and subsequent annealing. A crystallographic model of the martensite nucleation and the rearrangements β_1_→β_1_′ and β_1_→γ_1_′ are proposed based on the analysis of the observed tweed contrast and diffuse scattering in the austenite and the internal defects in the substructure of the martensite.

## 1. Introduction

The external temperature and force mechanical effects, as well as magnetic and electric fields, which provide for thermoelastic martensitic transformations (TMTs), allow one to actualize a number of unusual and extremely important physical phenomena in various materials. The single- or multiple-cyclically reversible shape memory effects (SMEs) and gigantic superelasticity and damping associated with TMT enable the classification of smart shape memory alloys (SMAs) into a special separate class of practice-important structural multifunctional materials [1,2,3,4,5,6,7,8,9,10]. Recently, it was found that SMAs are also distinguished by gigantic caloric effects, including magnetocaloric, electrocaloric, barocaloric, and elastocaloric, which are in demand in effective environmental thermo-refrigeration technologies [11,12,13,14,15,16,17,18,19,20,21,22,23].

The rapid development of modern equipment and technologies dictates the creation of such smart materials that can be practically used in a wide range of temperature-, power-, and other operating conditions. However, a significant disadvantage of polycrystalline smart materials, with the exception of binary alloys of titanium nickelide, is their low ductility and brittleness, which exclude the implementation of these unique effects not only in cyclic multiple, but even in a single application. Therefore, the problems of the optimum alloying and development of methods for plasticization of the polycrystalline materials with SMEs for their various industrial applications are becoming increasingly important, but remain unsolved.

Copper-based β SMAs, such as Cu-Al-Ni, Cu-Zn-Al, and Cu-Zn-Sn, are distinguished by their much lower cost, better thermal and electrical conductivity, and superior technological processability in comparison with, for example, alloys based on titanium nickelide [1,2,3,24]. Moreover, these β alloys in a single-crystalline state demonstrate excellent SME characteristics. However, in a usual coarse-grained (CG) state these polycrystalline alloys are also of extremely low plasticity, crack resistance, and fatigue life characteristics [2,24]. This does not permit the actualization of SMEs that the β single crystals are typical of.

The specific cause of the brittleness due to the operation of the mechanism of intergranular fracture typical of copper alloys that are metastable with respect to TMT is the low value of the modulus C′ = C_11_−C_12_/2 and, accordingly, the high anisotropy of the elastic modulus, A = C_44_/C′ (12–13 units) [25], whereas for elastic-isotropic low-modulus and ductile alloys of titanium nickelide, the value of A is correspondingly only 1–2 units [26,27,28,29,30,31,32,33,34,35]. The large elastic anisotropy at TMT leads to significant elastic stresses at the joints of martensitic packets and particularly at the grain boundaries, and their magnitude and localization at the boundaries are greater the larger the alloy grains are. The decrease in plasticity is aggravated by the grain-boundary chemical liquation and heterogeneous decomposition in these CG alloys—primarily at temperatures below the eutectoid decomposition boundary (T_ED_), which is close to 840 K [2]. Intergranular brittleness is one of the key reasons preventing the practical application of these SMAs.

At the same time, it has been established that a noticeable improvement in the strength- and plasticity-related characteristics of NiTi-based SMAs is achieved upon the formation of an ultra-fine-grained (UFG) structure [35,36,37,38,39,40,41,42,43,44,45,46,47,48,49,50]. The formation of the UFG structure is provided by advanced thermo-deformational technologies with employment of such methods of severe plastic deformation (SPD) as equal channel angular pressing (ECAP), high pressure torsion (HPT), high temperature pressing, multipass rolling, and drawing into strips, rods, or wire.

In our works [35,51,52,53,54,55,56,57] it was found that a radical decrease in the grain size during SPD and, accordingly, an increase in the length of the grain boundaries permits us to make the level of embrittlement of SME β-copper alloys lower. Any other methods of refinement of the grain structure of these alloys using alloying additives, heat treatment, rapid quenching, powder metallurgy, and a number of other corresponding methods turned out to be generally unsuccessful [58,59,60,61,62,63,64,65]. The aim of this presented work was to study (i) the structure of the Cu-Al-Ni-based SMAs and (ii) the effect of the SPD by means of HPT and subsequent annealing on the grain sizes, structural phase transformations, mechanical properties, and hardness.

## 2. Materials and Methods

The master alloys of nominal compositions Cu–14%Al–3%Ni (that at room temperature (RT) is in the austenitic state) and Cu–13.5%Al–3.5%Ni ((in wt.%) that is in the martensitic state) were melted out from the components Cu, Al, and Ni of high purity (99.99%). The chemical composition of the alloys, determined by the spectroscopy analysis, is given in Table 1. The alloys were subjected to hot smith forging at 1173–1273 K to the rod with the cross section of 20 × 20 mm^2^ and to water quenching from 1223 K after heating for 10 min. A number of samples of the alloys were subjected to repeated water quenching from 1273 K after preceding heating for 30 min. For the thorough refinement of the grain structure of the alloys we employed an HPT method under a pressure of 6 GPa at RT to 1, 5, and 10 revolutions in highly rigid Bridgman-anvil-type units (flat or with a cylindrical recess in the lower anvil. Specimens for HPT were made in the form of disks—10 mm in diameter in the case of flat anvils and 20 mm in diameter in the case of anvils with a cylindrical recess, 0.5 and 1.2 mm thick, respectively. The value of the true deformation (e) of specimen disks at half the radius was 4.65 or 6.0 units. Isochronous isothermal anneals of the HPT specimens were performed in the temperature range 373–873 K (in increments of 100 K) for 30 min. The critical temperatures of the start (M_s_, A_s_) and end (M_f_, A_f_) of the direct (M_s_, M_f_) and reverse (A_s_, A_f_) TMTs were determined in the course of the cyclic temperature measurements of the magnetic susceptibility χ(T) and electrical resistivity ρ(T), with a rate close to 5 K/min. The structure and the phase composition were investigated using the methods of X-ray diffractometry (XRD), optical metallography (OM), transmission- (TEM) and scanning electron microscopy (SEM), including EBSD analysis (of diffraction of back-scattered electrons), and MEA (energy-dispersive X-ray spectral microelement analysis). XRD studies were performed in the copper radiation Cu*K*α. The microscope TEM Tecnai G^2^ 30 (Hillsboro, OR, USA) was employed at an accelerating voltage of 300 kV; SEM Quanta 200 (Hillsboro, OR, USA), equipped with the system Pegasus, was employed at an accelerating voltage of 30 kV. Thin foils of Ø3 mm (in diameter) were prepared by the method of ion etching on the setup Fischione 1010 IonMill (Pittsburgh, PA, USA) by cutting out the 1/2 radius and subjecting it to grinding–polishing on a Metaserv 250 apparatus (Chicago, IL, USA). Vickers’s microhardness measurements (H_V_) were performed on a Micromet 5101 (Chicago, IL, USA) with a pyramidal diamond indenter at a load of 1 N (100 g). Tensile tests of small-sized flat specimens with a length of 10.0, thickness of 0.25, and width of 1.0 mm were performed at a special automated setup. Tensile tests of standard bulk specimens of Ø3 mm thickness were performed at an Instron 8862 setup (Buckinghamshire, UK).

## 3. Results and Discussion

### 3.1. Pre-Martensitic State

**Figure 1:**
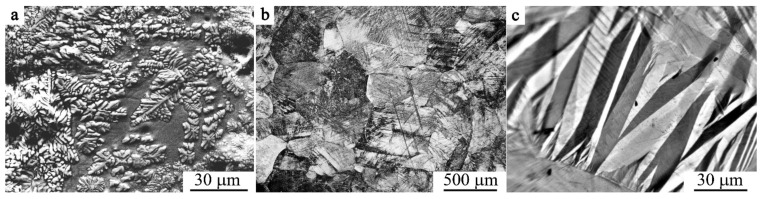
(**a**) SEM image and (**b**,**c**) OM images of the alloys (**a**,**c**) Cu–13.5Al–3.5Ni and (**b**) Cu–14Al-3Ni in the (**a**) as-cast, (**b**) austenite, and (**c**) martensite states.

As was determined, hot deformation via forging of the studied Cu-based alloys permits refining of austenite grains down to 0.5–1 mm [54,55]. However, the further subsequent cooling in air entails (i) the decomposition by the scheme β→β_1_+γ_2_ (at temperatures above the T_ED_ close to 840 K) and (ii) eutectoid decomposition via β_1_→α+γ_2_ (at temperatures below T_ED_) (Figure 1a), which is in good correspondence with the known data from [2,24,35,51,52,53,54,55,56,57,58,59,60,61,62,63,64,65]. However, quenching of the alloys after their hot forging allows us to prevent the eutectoid decomposition. At the same time, it is important to take into account that β-austenite at temperatures above T_ED_ and M_s_ successfully and successively experiences two transitions (disorder–order), namely, β→β_2_(B2)→β_1_(D0_3_). In this case, as is known, an inheritance of the long-range atomic order from the initial atomically ordered austenitic phase is provided by emerging martensite. The latter fact, as a consequence, determines the thermal elastic behavior of the martensite [1,2,3,4,5,6].

Figure 1b,c displays typical OM images of (b) the grain microstructure of β_1_-austenite and (c) the intragrain packet-pyramidal pairwise-twinned morphology of martensite of the quenched alloys (b) Cu–14Al–3Ni and (c) Cu–13.5Al–3.5Ni.

**Figure 2:**
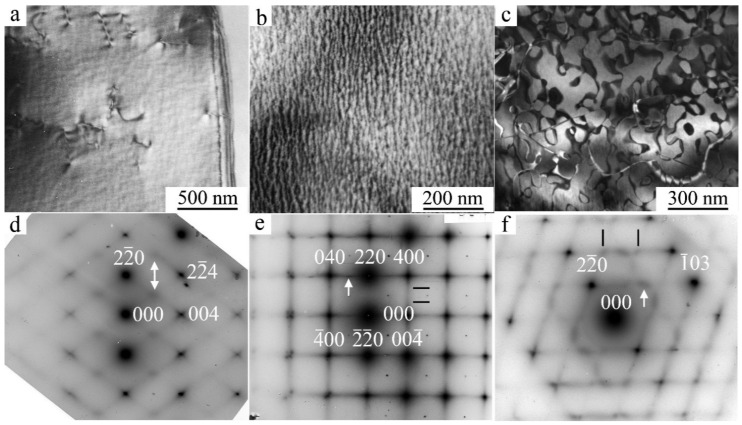
(**a**–**c**) Dark-field TEM images of (**a**,**b**) tweed contrast, (**c**) anti-phase boundaries, in the β_1_-austenite of the quenched alloy Cu–14Al–3Ni and (**d**–**f**) corresponding SAED patterns, with zone axis (**d**) [110], (**e**) [001], and (**f**) [331]; D0_3_. Observations at (**a**,**d**) 450 K, and (**b**,**c**,**e**,**f**)–RT.

**Figure 3:**
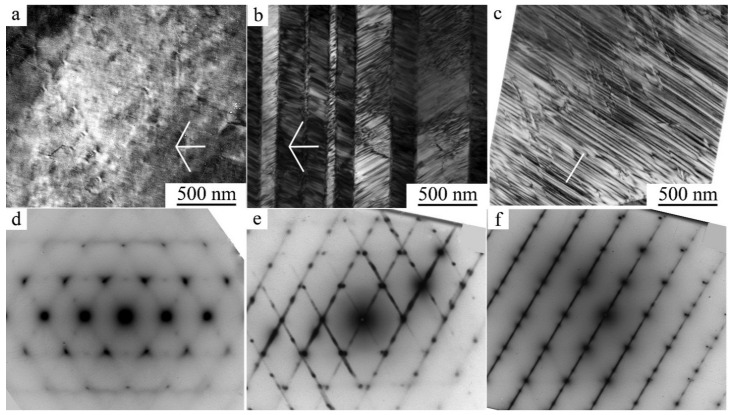
(**a**) Dark-field and (**b**,**c**) bright-field TEM images (**a**) of the tweed contrast in the β_1_-austenite, (**b**) twinned γ_1_′ martensite, and (**c**) β_1_′ martensite of the quenched alloy Cu–13.5Al–3.5Ni, and (**d**–**f**) their corresponding SAED patterns, with zone axis close to [111]; D0_3_. Observations (**a**,**d**) at 450 K, and (**b**,**c**,**e**,**f**)–at RT. In addition, white lines (**a**,**b**,**c**) denote the directions of the normals to (i) the planes of {111} type, (ii) the traces of stacking faults, and (iii) the micro twins of the martensitic crystals with the same habit, which is close to {111} D0_3_.

During TEM studies, in the bright- and dark-field images of quenched austenite one can observe the so-called tweed contrast (Figure 2a,b and Figure 3a) and anti-phase domain boundaries (APB) (Figure 2c). Through such examinations, an intricate regular visual image of non-radial diffuse scattering is revealed in selected area electron diffraction (SAED) patterns, and its reconstruction in the form of a reciprocal lattice leads to a successive revealing of the arrangement of the flat diffuse {111}* (walls) that pass through all the nodes hkl, excluding the central node 000 [6,66].

The distribution of the intensity of diffuse scattering in these {111}* walls varies heterogeneously and naturally, depending on the position in the reciprocal lattice. A more intense diffuse scattering is observed in the vicinity of any fundamental reflections, rather than in the space between them (Figure 2 and Figure 3). According to the diffraction theory, the intensity of diffuse scattering is determined by the relation I ~ |A(k)|^2^ (g e^(k)^)^2^, where A(k) is the displacement waver amplitude, k is the wave vector, e^(k)^ is the polarization vector, and g is the diffraction vector [6,66]. The scattering is absent (except for the cases of double diffraction) if the g is directed in parallel to the plane of a diffuse wall (i.e., the vector g lies perpendicular to e^(k)^ in the given planes passing through the center of the reciprocal lattice). The most intense diffuse streaks in the reciprocal lattice are located parallel to k || <110>*, and e^(k)^ || <$\overset{-}{1}$10>*. A specific feature of the diffuse scattering effects with increasing diffraction angle is the preservation of their sufficiently high intensity in comparison with decreasing intensity of Bragg fundamental reflections. Such scattering occurs at the temperature of observation in situ above M_s_ by 100–150 K (Figure 2 and Figure 3).

**Figure 4:**
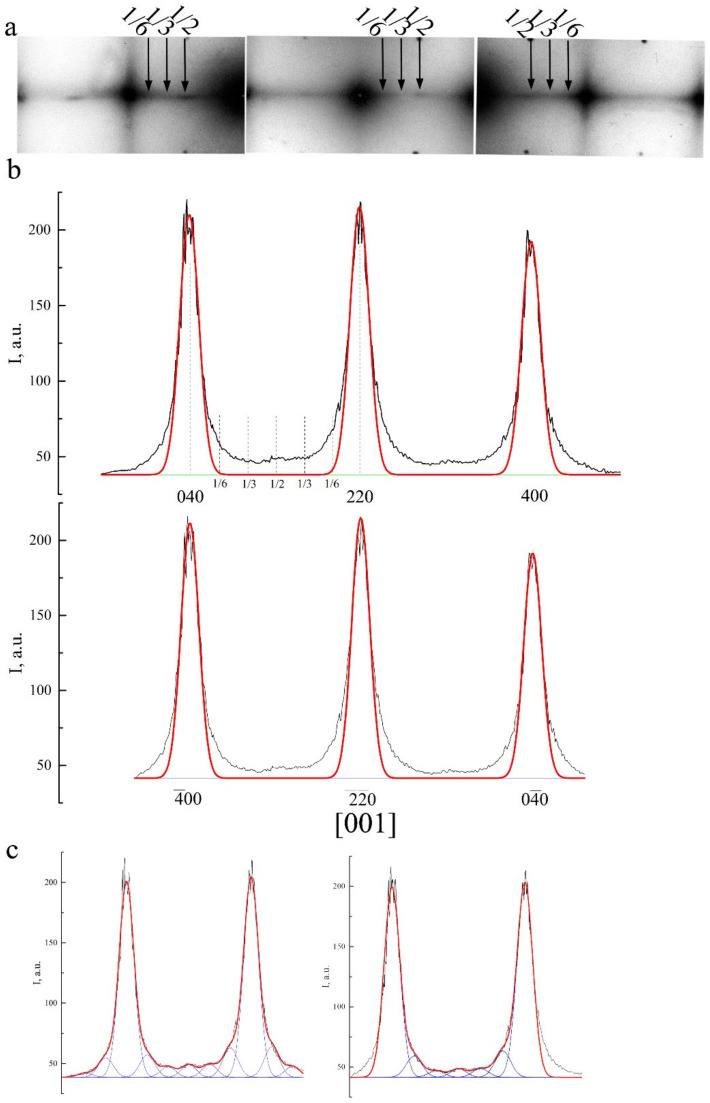
(**a**) Fragments of SAED pattern with zone axis [001]. (**b**) Intensity profile when scanning the diffuse scattering between the fundamental reflections of types 040–220–400 or −400–−2-20–0−40. (**c**) Intensity profile along the diffuse streaks with calculated profiles for satellites of type 1/6 <220>, 1/3 <220>, 1/2 <220>.

**Figure 5:**
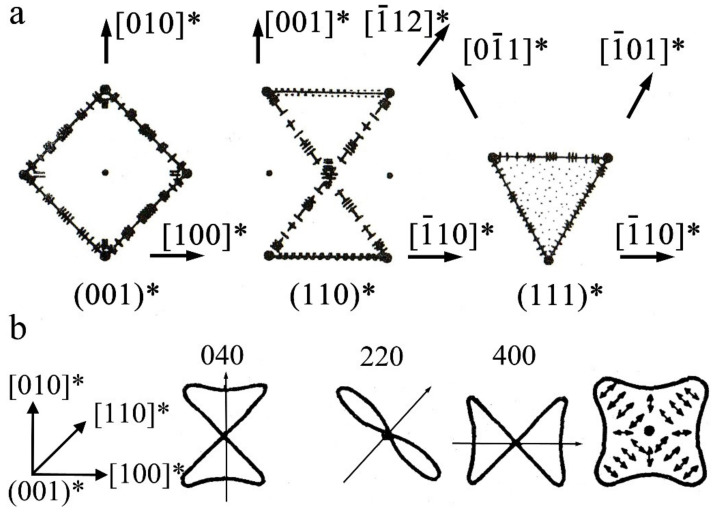
(**a**) Spectra of waves of atomic displacements in the form of the planar cross sections (001)*, (110)*, and (111)* of reciprocal **k** space and (**b**) in vicinity of reciprocal lattice nodes in the (001)* planes.

**Figure 6:**
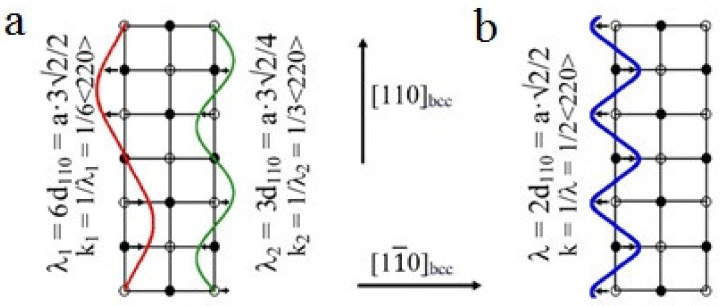
Schemes of shuffling offsets type of 1/6<220>**_k_**<1–10>**_e_** and 1/3<220>**_k_**<1–10>_e_ (**a**) and 1/2<220>**_k_** <1–10>_e_ (**b**) that provide transformations cubic lattice to D0_3_ or L2_1_.

As the temperature approaches M_s_, the intensity of the diffuse streaks along <112>* and particularly <110>* (according to qualitative and quantitative estimations) increases gradually and, apart from this, which is very important, at these streaks one can see the increase in intensity of the extra reflections (called satellites) at the positions in the space of reciprocal lattice close to 1/2<220>*, 1/3<220>*, 1/6<220>*, 1/2<422>*, and 1/3<422>*. The appearance of such satellites is due to the coexistence of different lattice waves of displacements of k_n_ atoms in a crystal that is metastable with respect to the shear transition. The diffraction pattern of such a crystal with a single wave of type k_n_ is described by the vector relation g = g_hkl_ + Σp_n_·k_n_, where k_n_ is the wave vector from the star of the k_n_ vector, {k_n_}, p_n_ are integers. In this case, it is possible to register paired satellites of the first order (±k). In highly symmetric crystals, multi-ray states are realized, including, for example, two rays of the same set. Therefore, if we denote k_1_ = 2/3 2/3 0, k_2_ = 2/3 0 2/3, then k_1_ + k_2_ = 4/3 2/3 2/3. This resulting vector (in units of 2π/a) in the reduced Brullien zone corresponds to the vector k′ = 200−(4/3 2/3 2/3) = 2/3 −2/3 −2/3 and characterizes the diffraction vectors as second order. In other words, satellites of the type 1/3<222>*, 2/3<222>*, and 1/3<422>* that are significantly weaker than the 1/3<220>* satellites of the first order have an interference origin and are second-order satellites. This, in particular, means that the ω-shaped displacements in the D0_3_ and L2_1_ lattices, the presence of which was assumed in [66], can be obtained by the interference of waves 1/3 <220>_k_<1–10>_e_. In addition, it should be noted that the position of the satellites of type 1/2 <422>* coincides with the punctures of the Ewald sphere by diffuse streaks along <110>* (see Figure 2d and [66]). Thus, only satellites of the type 1/2<220>*, 1/3<220>*, and 1/6<220>* should be considered the effects of independent diffuse scattering together with continuous diffuse scattering along <110>* (see Figure 4). The black profile lines represent the experimental profiles of intensity that were measured using the program Digital Micrograph when processing SAED patterns. The red profile lines represent the profiles of intensity calculated by employing a Gauss Function within the framework of the program Origin for fundamental reflections. The blue lines represent the calculated profiles for the satellites of the types 1/6 <220>, 1/3 <220>, and 1/2 <220>. Typical features of (i) the observed diffraction tweed contrast and (ii) diffuse electron scattering, i.e., their periodic “reoccurrence”, regular attenuation, and amplification, make it possible to describe them (namely, i and ii) by certain spectra (see Figure 5) of the transversal and longitudinal vibration waves in the k-space of reciprocal lattice, which are characterized by the wave (k) and polarization (e^(k)^) vectors, and, correspondingly, to identify them with the localized waves of atomic displacements in the space of crystal, which sufficiently periodically distort, on average, the original crystal lattice (Figure 6). The projections of vectors e^(k)^, shown in Figure 5 by streaks or arrows, are characteristic of the waves of enhanced amplitude and, consequently, of intense scattering. The interpretation of the data obtained gives an opportunity to construct a physical model of the real metastable microstructure and its evolution and assess its possible role in the nucleation mechanism of TMT in low-modulus Cu-Al-Ni alloys.

As is known, in the BCC alloys the scattering between reflections in the form of diffuse walls {111}* is stipulated by short-wave acoustic (predominant-in-the-spectrum) vibrations of the non-correlated displacements of closely packed (along <111>) chains of atoms relative to each other. At pre-martensitic softening of elasticity moduli, particularly of C’ [6,25,67], the amplitudes and correlations of such specific linear <111> defects of atomic displacements gradually grow in the close-packed planes {110} (Figure 6). If the correlations of atomic displacements in these planes are greater than correlations between the planes themselves relative to each other, then the diffuse scattering has the form of solid (continuous) streaks along <110>*. Judging by the character of tweed contrast, such atomic displacements are localized within the volume of nano regions, whose distorted structure and symmetry can be described by the short-range order of atomic displacements (SOD) [34,35,66]. 

When the alloys are cooled down below a certain temperature T_IncS_ (at the stage of weakly Incommensurate Satellites), the diffraction pattern commences to be characterized by the appearance of satellites–in the main of the type “1/6”, “1/3” and/or “1/2”, which correspond to long-period modulation (LPM) nanostructures: (for brevity)–LPM-1 (for the satellites of type “1/3”) and LPM-2 (for the satellites of type “1/2”) [35]. The satellite-related stage can be considered an independent state that replaces SOD domains and is characterized by quasi-stable intermediate LPM shear substructures. Since all crystallographically equivalent variants of the distortion-induced (orientation-related and anti-phase) LPM nanodomains located statistically along the volume of the austenitic phase exist, the structure of such alloys on average retains the original cubic symmetry. According to the diffraction-provided data, the internal distortion and local symmetry of the LPM domains differ from the original one and obviously approach the structure of the future martensitic phases as much as possible while maintaining a coherent connection under the conditions of the specifics of the progressive local instability of the crystal lattice of the austenitic phase and its anharmonism [6,35]. In the experiment, this structure-related nucleation mechanism of the TMT is confirmed, firstly, by the fact that the reflections in the SAED patterns from martensite crystals of the β_1_′ and γ_1_′ phases are located precisely in the positions of the satellites of 1/3 and 1/2 types (see Figure 3). Secondly, at the nucleation and growth of these phases, there emerge a large number of planar chaotically disposed stacking faults (SF) coplanar to the basal plane of (001) type for both of the martensitic phases β1′ and γ_1_′. In this case, one can observe specific characteristic features of the contrast (Figure 3b,c) and diffuse scattering in the form of distinct narrow continuous streaks passing through Bragg reflections (Figure 3e,f).

**Figure 7:**
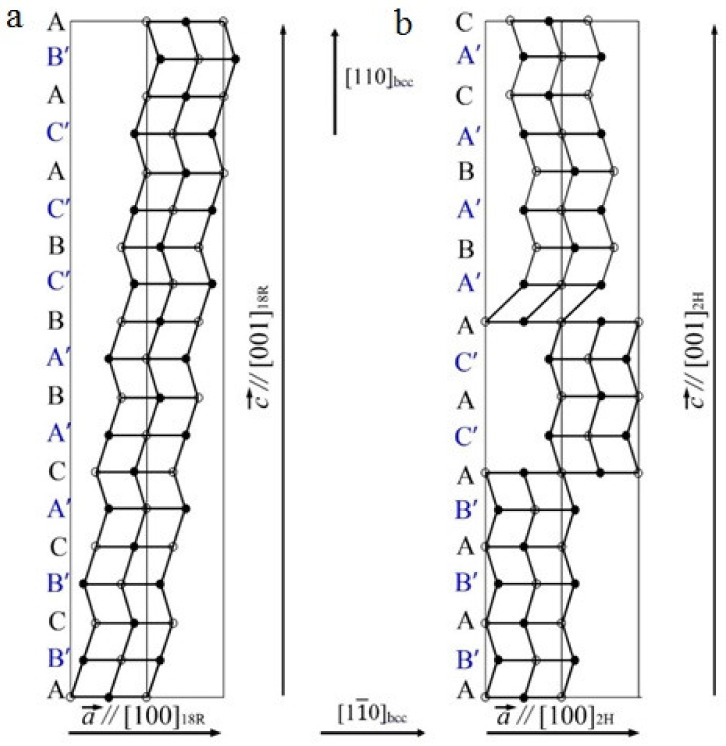
Schematics of crystal lattice rearrangement of type (**a**) D0_3_→18R and (**b**) D0_3_→2H martensite.

In the modern non-classic crystal-structural description, LPM domains are by themselves special-type nano nuclei (with the structure non-identical to that characteristic of either the austenite phase or would-be martensite phases) and can play the role of real physical centers of nucleation of martensite crystals. Thus, at a certain synchronization between the uniform longitudinal distortion of <100>**_k_**<100>**_e_** Bain type and the transversal static waves of atomic displacements describing the structure of the nano domains LPM-1 by the two waves of 1/6<220>**_k_**<1–10>**_e_** and 1/3<220>**_k_**<1–10>**_e_** type (Figure 5 and Figure 6), the structure of the β_1_′ martensite of 18R type in the studied alloys can be obtained (see Figure 6a and Figure 7a). Additionally, the combination between (i) the uniform longitudinal of <100>**_k_**<100>**_e_** Bain distortion and (ii) the mode of the periodic shuffling displacements of the type 1/2<220>**_k_** <1–10>**_e_**, which form the structure of the nano domains LPM-2, crystallographically presets the trend towards the rearrangement β_1_→γ_1_’ (Figure 6b and Figure 7b). Apparently, in this case, violation of the ideal stacking of atoms along the basal plane (001) is feasible, and this entails the occurrence of various disturbances in the form of stacking faults (Figure 7). 

**Figure 8:**
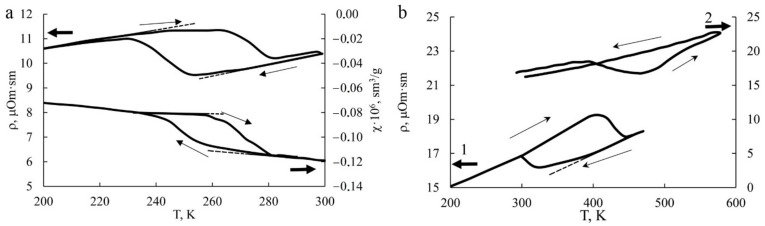
(**a**) The temperature dependences ρ(T) and χ(T) of the Cu-14Al-3Ni alloy after quenching from 1223 K in water in the measurement cycle 300 K→LN→300 K; and (**b**) temperature dependences of ρ(T) of the Cu-14Al-3Ni alloy after HPT in the measurement cycles 300 K→LN→470 K→300 K (curve 1) and 300 K→573 K→300 K (curve 2) (LN is an abbr. for liquid nitrogen). The dotted line shows the pre-transition deviations from the linear dependence of ρ(T) and χ(T) interval of 20–25 K; inside hysteresis loops.

**Figure 9:**
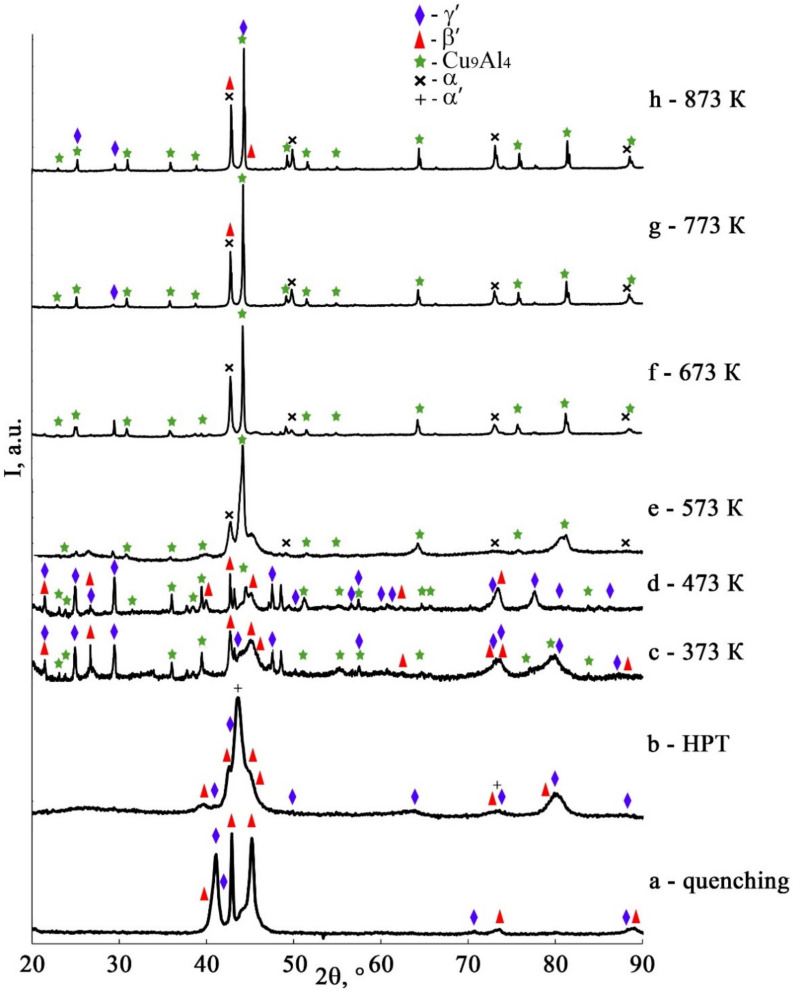
XRD spectra for the alloy Cu–14Al–3Ni after its (**a**) quenching, (**b**) HPT of 10 rev, and (**c**–**h**) HPT of 10 rev + annealing at 373 K–873 K, 30 min. Temperatures of measurements: (**a**) 200 K, (**b**–**h**) RT.

Interestingly, when measuring the temperature dependences ρ(T) and χ(T) in the thermal cycles (cooling from RT to 90 K–heating to RT), not are only the hysteresis loops of the TMT are determined, but the pre-martensitic deviations of the dependences from linearity of the curves ρ(T) and χ(T) can also be found, despite their being a bit narrower in an interval of the temperatures 20–25 K, which follows from the data of the TEM analysis, obviously being more sensitive (Figure 8). The temperature dependences for the Cu-14Al-3Ni alloy shown in Figure 8b demonstrate the TMT hysteresis loops when measured in other thermal cycles cooling–heating (curve 1) or heating–cooling (curve 2). The temperatures determined by the two-tangent method are shown in Table 2 and are consistent with the XRD phase-composition data (Figure 9). 

As has already been noted, on the one hand, low values of elastic modulus, and on the other hand, the possibility of deformation-induced TMT, both these circumstances determine the unique specific features of the mechanical behavior of metastable alloys. In the next section, we will consider the results of applying SPD to the alloys studied in this work in order to create a nanocrystalline UFG state in them.

### 3.2. Structure, Phase Composition, and Mechnical Properties of SPD Alloys

According to XRD data, in the course of cooling below the temperature M_s_ in the studied quenched β_1_ Cu-Al-Ni alloys, two martensitic phases indicated as β_1_′ (of 18R type) and γ_1_′ (of 2H type) are formed (see Figure 9, curve (a)). It is established that an HPT of 10 revs at RT entails the formation of these alloys of a mixture of the three deformation-induced martensitic phases α′, β_1_′, and γ_1_′ (Figure 9, curve (b)) in accordance with [68]. The Bragg reflections detected in this case are significantly broadened (with a half width up to 2 degrees) and coincide with the most intense diffraction lines from these martensitic phases.

It is important to note that the hysteresis of the TMT (ΔT) in the HPT-treated alloy increases more than three times with a noticeable increase in all critical temperatures. According to the data of XRD analysis of the HPT-treated alloy subjected to annealing at 373 and 473 K, in the alloy, the martensitic phases β_1_′ and γ_1_′ are preserved and the γ_2_–Cu_9_Al_4_ phase of aging appears (see Figure 9, curves c, d). An annealing at temperatures higher than A_f_, namely, 573–773 K leads to the eutectoid decomposition of β_1_ austenite into the phases (α + γ_2_) (see Figure 9, curves e–g). Finally, the annealing at 873 K causes the decomposition of austenite with precipitation of the γ_2_ phase (Figure 9, curve h). Further cooling of the HPT-treated alloy, down to RT, is accompanied by the TMT in the retained β_1_ matrix. Therefore, in XRD analysis, β_1_-phase reflections, unlike martensitic reflections, are not detected (see Figure 9).

**Figure 10:**
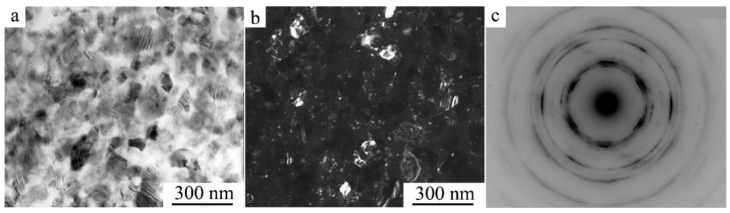
(**a**) Bright- and (**b**) dark-field TEM images of the microstructure and (**c**) the corresponding SAED pattern for the Cu–14Al–3Ni alloy after its quenching from 1223 K and HPT of 10 revs.

**Figure 11:**
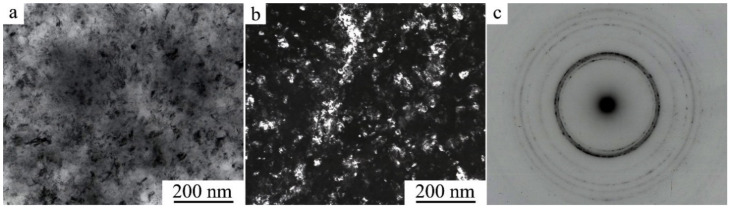
(**a**) Bright- and (**b**) dark-field TEM images of the microstructure and (**c**) the corresponding SAED pattern for the Cu–13.5Al–3.5Ni alloy after its quenching from 1223 K and HPT of 10 revs.

TEM investigations have shown that as a result of HPT when increasing the number of revolutions from 1 to 10 (and, correspondingly, the degree of deformation) in both alloys, a (progressively) more uniform nano-grained martensitic structure is formed, which is characterized by a ring-wise distribution of Bragg reflections in the SAED patterns (see Figure 10 and Figure 11).

According to the data of quantitative analysis of the bright- and dark-field TEM images taken from the alloys after their HPT of 10 revs, the sizes of the observed randomly oriented structural fragments that are most frequently occurring vary within the limits from 10 to 80 nm and are on average 30 nm. It is seen that the larger (in size) of them has plate-like nano twins. Indexing of SAED patterns has shown that the nanocrystalline structure formed in the alloys contains predominantly three martensitic phases (α′ + β_1_′ + γ_1_′). The ring distribution of the reflections implies the presence of both small- and large-angle misorientations of the martensitic nanophases that make up the ultrafine-grained (UFG) structure.

**Figure 12:**
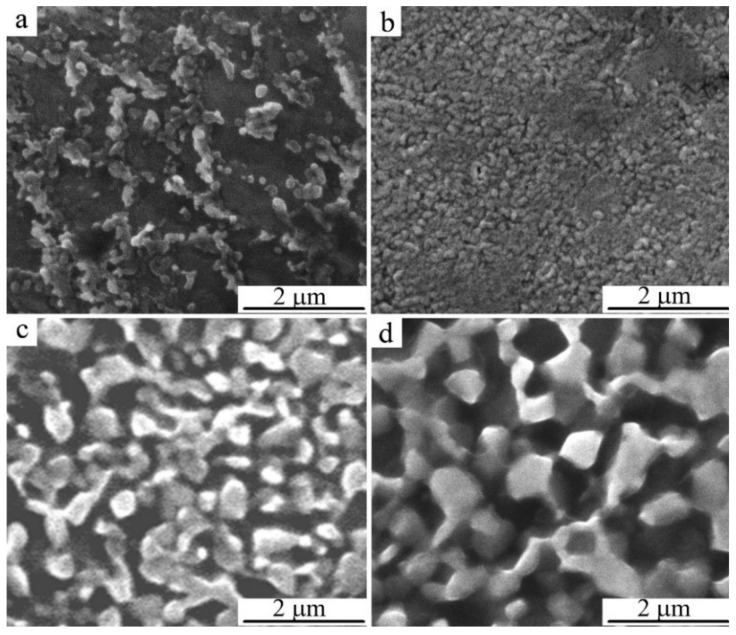
SEM images of the microstructure of the Cu-14Al-3Ni alloy after the HPT of 10revs and anneals for 30 min at temperatures of (**a**) 373, (**b**) 673, (**c**) 773, and (**d**) 873 K.

**Figure 13:**
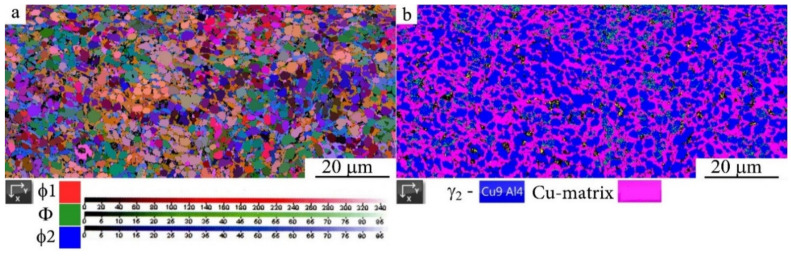
(**a**) Map of the disorientation of the structure in Euler angles and (**b**) phase map of image quality in EBSD mode for HPT Cu-14Al-3Ni alloy of *n* = 10 revolutions, and annealing at 873 K, for 60 min.

We have studied the effect of the temperature of annealing on the microstructure of the HPT-treated alloys using SEM microscopy and revealed the following. Anneals of martensite at 373 and 473 K did not lead to noticeable size-morphological changes in the martensitic UFG structure formed as a result of HPT. However, anneals in the austenite condition (in the temperature range from 573 to 873 K) have provided a noticeable growth of globular grains in the process of formation of UFG states in the HPT-treated alloys (Figure 12). Employment of the orientational EBSD method has permitted us to establish the specific mechanism of decomposition in the HPT alloys, as a result of which one can observe the formation of a UFG structure of alternating nanophases (see, for example, Figure 13).

**Figure 14:**
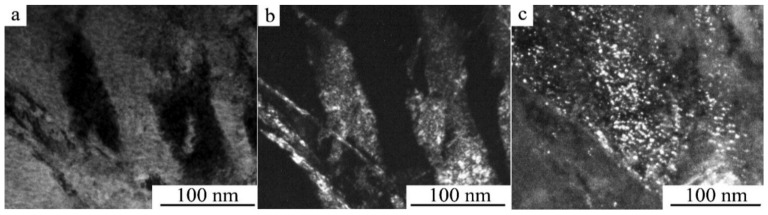
(**a**) Bright- and (**b**,**c**) dark-field TEM images of the aging martensite microstructure in the coarser grain of the Cu-14Al-3Ni alloy after the HPT of 10 revs and isochronous anneals (for 30 min) at 373 K.

Low-temperature annealing below M_s_ initiates pro-eutectoid homo- and heterogeneous decomposition of martensite in the HPT-treated alloys with precipitation of an aluminum-enriched γ_2_ nano phase (Figure 14). Namely, the depletion of matrix of aluminum by 1–2 at.% causes the destabilization of austenite and an increase in the critical temperatures of the TMT by 70–150 K (see Figure 8 and Table 2).

**Figure 15:**
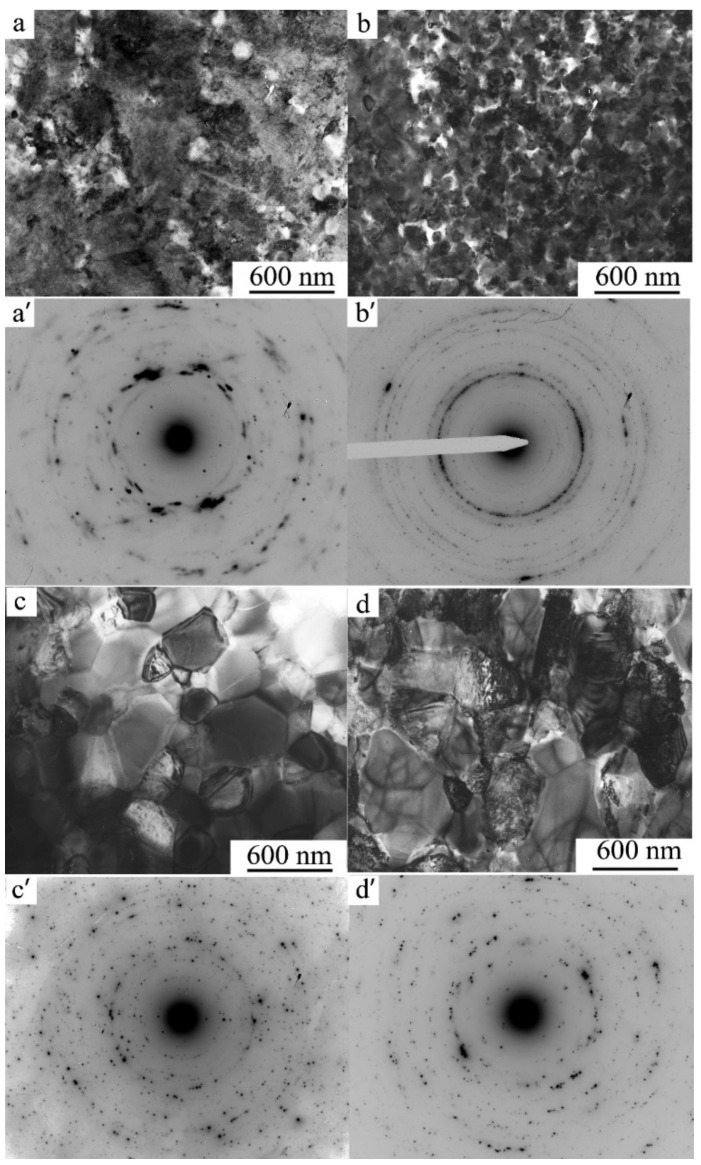
(**a**–**d**) Bright-field TEM images of microstructure and (**a**’–**d**’) corresponding SAED patterns of the Cu-14Al-3Ni alloy after the HPT of 10 revs and isochronous anneals (for 30 min) at temperatures of: (**a**,**a**’) 573 K, (**b**,**b**’) 673 K, (**c**,**c**’) 773 K, and (**d**,**d**’) 873 K.

**Figure 16:**
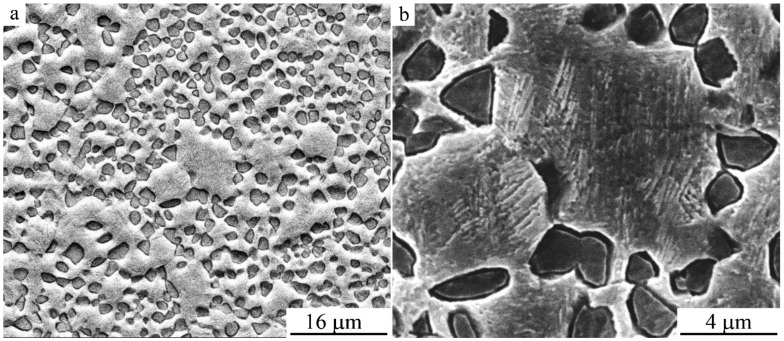
SEM images of the microstructure of the Cu-14Al-3Ni alloy after the HPT of 10 revs and annealing at 1073 K for 10 s at (**a**,**b**) different magnifications.

A study of the alloys in the state of β_1_-austenite after HPT of 10 revs and high-temperature anneals was also performed via TEM methods (Figure 15 and Figure 16). According to the data of bright-field TEM investigations, it was confirmed that in the UFG β_1_ matrix, in the course of annealing, there was a formation and growth of the globular nano crystallites of (in the main) three phases, namely, the β_1_, α, and γ_2_ phases, judging from the results of indexing of corresponding SAED patterns (the values of the average size <d_G_> of the grains–crystallites are given in Table 3). We have a right to draw a conclusion that annealing at 573 K triggers in the reverted β_1_-austenite a combined reaction of the process of recrystallization and eutectoid (α + γ_2_) decomposition. In this case, with a background of coarser recrystallized grains, the smaller (comparative) grains–crystallites are still distinguished by the intense deformation-related contrast and they remain virtually unaltered in their sizes. On average, the value of <d_G_> is close to 100 nm (see Figure 15a’). The reflections in the corresponding SAED patterns still exhibited a continuous (solid) ring distribution (Figure 15a’).

An increase in the annealing temperature to 673 K led to almost complete primary recrystallization of the reverted β_1_-austenite and to the formation of a (β_1_ + α + γ_2_) nano-triplex structure in the composition of a homogeneous UFG austenite at an increased <d_G_> value close to 150 nm. The boundaries of grains–crystallites remained rounded and distorted, and the structure still retained an increased number density of defects (Figure 15b). Intensive enlargement of the α and γ_2_-phase globules formed in β_1_-austenite at <d_G_ > close to 350 nm occurred at a higher isochronous annealing temperature of 773 K (Figure 15c). After annealing at 873 K, the <d_G_> for the β_1_ and γ_2_ phases is already close to 400 nm (Table 3, Figure 15d). Imaging of the boundaries of grains–crystallites of a nano-duplex (β_1_ + γ_2_) structure, which are inherited by martensite, becomes even more distinct, and one can observe a contrast between the twins in martensite. More “sharp” point reflections in the SAED patterns indicate a significant relaxation of the internal stresses and interfacial distortions in the UFG structure formed by the precipitated phases and martensite. The ring-shaped visual character of the less uniform distribution of point reflections in the SAED patterns of HPT-treated alloys after annealing at 673–873 K indicates the preservation of small-angle and large-angle random misorientations of the resulting globular grains–crystallites in the alloy (see Figure 15a’–d’). Another type of a bimodal single-phase UFG structure, the martensite UFG structure of HPT-treated alloys of the Cu-Al-Ni system, which was formed during short-term thermal action in the course of high-temperature annealing at 1073 K; for 10 s, is shown in Figure 16. An increase in the annealing time causes accelerated grain growth, since in this case the annealing temperature of 1073 K significantly exceeds T_ED_, which is close in this case to 840 K, and this time the increase is inexpedient, as it leads to the softening of the alloy.

Thus, in the result of these TEM investigations it is established that anneals, starting from the temperatures above A_f_, lead to recrystallization of the reverted β_1_-D0_3_ austenite in the HPT-treated alloys Cu–14Al–3Ni and Cu–13.5Al–3.5Ni. In this case, sufficiently homogeneous UFG states can be obtained, the average grain sizes of which increase after annealing at 573–873 K (30 min) in the range from 30 to 400 nm or reach 3.5 μm after short-term annealing at 1073 K, 10 s (Table 3).

In all cases, the obtained XRD and SAED patterns have shown the presence of γ_2_-phase reflections, both after low-temperature (below M_s_) annealing at 373–473 K, when the HPT-treated alloy has experienced partial decomposition of martensite with the precipitation of disperse (fine) γ_2_ particles, and after high-temperature annealing as a result of eutectoid (α + γ_2_) decomposition of austenite at 573–773 K or its (of austenite) pro-eutectoid (β_1_ + γ_2_) decomposition at 873 K. If the matrix austenitic β_1_ phase was preserved after annealing, which is the case for all the selected regimes of treatment, the subsequent cooling to RT was accompanied by TMT.

**Figure 17:**
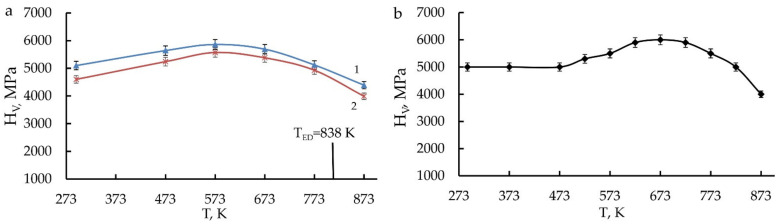
Dependence of the microhardness H_V_ of the HPT-treated alloys (**a**) Cu–14Al–3Ni (curve 1–quenching from 1223 K, curve 2–quenching from 1273 K) and (**b**) Cu–13.5–3.5Ni on the temperature of annealing for 30 min.

Data of measurements of Vickers hardness (H_V_) at the 1/2 of radius of the specimen disks of the HPT-treated Cu-14Al-3Ni and Cu-13.5Al-3.5Ni alloys in dependence of the quenching and temperature of annealing are listed in Table 4 and Table 5 and shown in Figure 17. It is established that H_V_ increases after HPT as the temperature of annealing rises, but H_V_ after double quenching remains slightly lower than after single quenching.

After their annealing at 573–673 K, the HPT-treated Cu-Al-Ni alloys had maximum H_V_ values, up to 6000 MPa after single quenching from 1223 K and 5550 MPa after repeated quenching from 1273 K. The HPT increases H_V_ by 1000 MPa in comparison with H_V_ in the initial quenched austenite condition. The repeated (second) quenching causes the refinement of grain and a better homogenizing of the solid solution of alloys than single quenching; however, as a consequence, this has led to slightly lower values of H_V._

**Figure 18:**
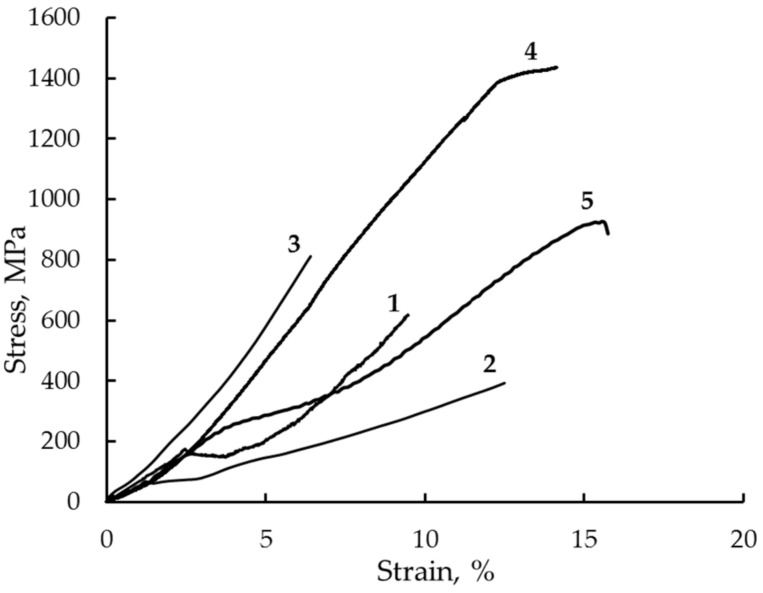
Stress–strain curves taken in tensile tests of the Cu-14Al-3Ni alloy after different deformation–temperature treatments: (1) forging + quenching from 1223 K; (2) repeated quenching from 1273 K; (3) HPT, *n*=10 rev, at 293 K; (4) HPT, *n* = 10 rev, at 423 K; (5) HPT, *n* = 10 rev, at 293 K + 1073 K, for 10 s.

The results of measurements of the tensile mechanical properties at RT of the Cu-14Al-3Ni alloy are shown in Table 6 and Figure 18. The tests showed that the quenched coarse-grained alloy had an ultimate tensile strength (σ_u_) of 620 MPa, a critical stress of martensite shear (σ_M_) 160 MPa, and a relative elongation to failure (δ) of 7%. The second quenching of the alloy with some softening due to the creation of a fine-grained state led to an increase in δ up to 11%. In the strengthened UFG alloy, subjected to HPT at RT, the value of δ decreased to 4%, and the destruction was brittle, without the formation of a neck. The plateau of phase fluidity in this case was absent in contrast to the cases of the quenched coarse-grained or the fine-grained alloy (where ε_M_ = 2%). The rise of the temperature of HPT of n revs to 423 K (by 130 K greater than RT) led to extremely high deformation-induced strengthening of the Cu-14Al-3Ni alloy and to a considerable growth of δ, whose value amounted to 12%. Therefore, in the HPT-treated alloy, the highest mechanical characteristics were achieved in complex. Thus, the yield strength has amounted to 1400 MPa, and the ultimate strength to 1450 MPa at a sufficiently high δ to failure (12%). Considerable changes in the mechanical properties of the alloy were observed after (i) HPT of 10 revs and (ii) isothermal annealing in the temperature range from 573 to 1073 K (see Table 6). All specimens are characterized by grain size conservation within the UFG structure.

### 3.3. Fractographic Study

**Figure 19:**
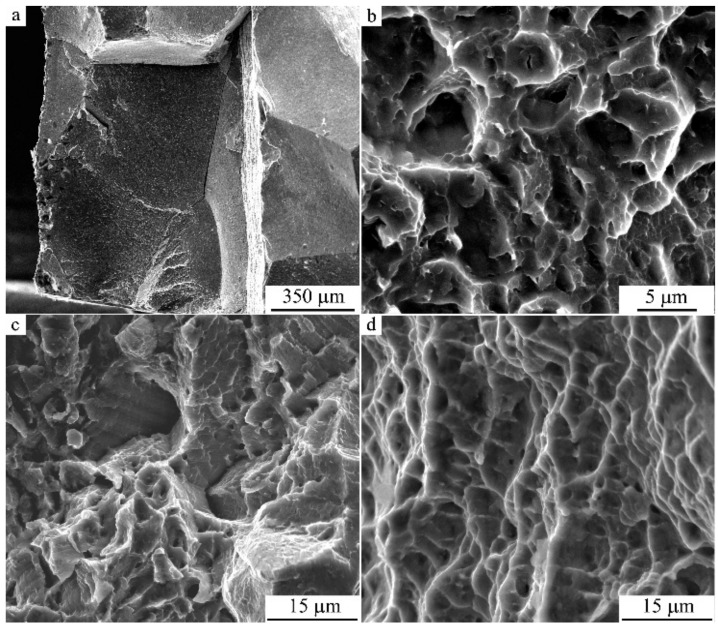
(**a**–**d**) SEM images of fracture surfaces of the specimens of Cu-14Al-3Ni alloy after (**a**) hot-forging and quenching (HFQ); (**b**) HFQ + HPT, *n* = 10 revs; (**c**) double quenching (DQ), with second Q from 1273 K; and (**d**) DQ + HPT, *n* = 10 revs + uniaxial tensile treatment at 423 K.

Fractographic investigations of the pattern of failure of the specimens after tensile tests showed that in the initial hot-forged quenched CG alloy the destruction of specimens took place in a brittle manner, predominantly via cleavage along the grain boundaries and large-scale packets of martensite crystals (Figure 19a). A nano-sized grain–subgrain structure after HPT of 10 revs has revealed the change in the type of fracture and the character of destruction of specimens (Figure 19b). In the latter case, on the fracture surface, there were many centers of localization of deformation with the formation of small flat dimples and, accordingly, low ridges of separation on the fracture surface. This, as a rule, is typical of the ductile (tenacious), intra-grain mechanism of failure of the alloys with low energy. However, the average size of dimples amounted to 2–5 μm, which was two orders greater than that of the elements of a UFG structure of the HPT-treated alloys. This circumstance testifies to the operation of the special intercrystalline mechanism of the fracture of their destruction, apparently along high-angle boundaries of the UFG structure, since in this case the size of cellular fragments (dimples) of separation during ductile–brittle fracture became two orders of magnitude smaller in comparison with the size of grains and regions of brittle cleavage in the initial coarse-grained alloy. This ultimately determined, in a number of cases, the increased ductility of the UFG alloys preliminarily subjected to HPT and consequent annealing (Figure 19b–d).

Therefore, it has been established that SPD of the eutectoid Cu-Al-Ni alloys with SMEs is, in certain conditions, an effective method of radical refinement of their grain structure and, as a consequence, attaining the appearance of plasticization. In the present work we have shown that an HPT to the large plastic deformations allows us to attain mechanically induced TMT and the concurrently evolving formation of a homogeneous UFG structure of martensite in metastable austenitic Cu-Al-Ni alloys, which is the case in both studied alloys. The UFG structure is generally characterized by high hardness, strength, as well as by a higher stability of martensitic phases with respect to the case of reverse TMT. The critical temperatures of the TMT of the alloy Cu–14Al–3Ni subjected to HPT of 10 revs have exhibited an increase in value: M_s_ and M_f_ by 70 K, and A_s_ and A_f_ by 160 K, (see Table 2). This allowed us to draw a conclusion that the increase in the temperatures of reverse TMT in the cycle of the heating and subsequent cooling when measuring ρ(T) is stipulated in the main, according to the data of phase analysis, by the precipitation effect of the γ_2_–Cu_9_Al_4_ phase (at the boundaries and inside the volume of martensite), which is responsible for both the dimensional and the chemical stabilization of martensitic grains–crystallites, the latter due to their depletion of aluminum and enrichment of copper (by 1–2 at.%, according to the data on M_s_ and A_f_). In this case, a known concentration dependence of the TMT temperatures on the composition in copper and aluminum [35] gives one the possibility of determining the chemical composition of the β_1_ matrix of quenched alloys of the system Cu–Al–Ni.

On the other hand, a number of specific features of the fine structure and phase composition of the alloys after HPT and isothermal annealing were revealed in combined analysis by the XRD, TEM, SEM methods, and measurements of ρ(T). It is established that at lower temperatures of annealing (373–473 K), namely, below A_f_, in the HPT-treated alloys one can find the preserved martensitic phases. In this case, the appearance of the reflections of the γ_2_ phase–“phase of ageing”, and the disappearance of the reflections of the strain-induced α’ phase, apparently transforming into β_1_′ martensite, are recorded. On the contrary, an annealing at higher temperatures, namely, above A_f_, leads to the disappearance of martensitic phases due to reverse TMT to a UFG austenite. In this case, at annealing below T_ED_ the reverted austenitic β_1_ phase is liable to experience a eutectoid (α + γ_2_) decomposition into a globular UFG nano-triplex structure, and at annealing above T_ED_ the precipitation of the γ_2_ phase with the formation of a nano-duplex (β_1_ + γ_2_) structure is expected. At quenching to RT or upon deformation, the appearance of TMT is possible.

It is important that post-deformation anneals of HPT-treated alloys have entailed the formation of structures capable of exhibiting SME and phase yielding in a wide range of stresses sufficient for the flow of martensite, namely, σ_M_ from 50 to 450 MPa (Table 6). Overall, anelastic deformation, including the formation of martensitic crystals, detwinning, and their reorientation in the direction of acting forces, provides an essential contribution to the ability of alloys to deform plastically. Thus, for instance, the alloy Cu–14Al–3Ni after HPT of 10 revs and short-term annealing at a temperature of 1073 K for 10 sec had δ of a relatively moderate value (13%), which was due to the combination of the values of the phase fluidity ε_M_ and duration of long-term stage of subsequent plastic deformation of martensite (Table 6). After attainment of the value σ_u_ = 900 MPa, there was a localization of the deformation with the development of a small reduction in the cross section of the neck.

## 4. Summary and Conclusions

In the present paper, two ternary SM alloys of close composition based on the Cu-Al-Ni system were chosen for investigation, namely, Cu-14%Al-3%Ni alloy in an austenitic state at RT, and Cu-13.5%Al-3.5%Ni alloy in a martensitic state at RT. 

A detailed investigation of the fine structure, morphological features, and physical properties of austenite and martensite in the alloys after their quenching, SPD, and consequent annealing was carried out. Tensile mechanical properties of the alloys in the coarse, fine-grained, and ultrafine grain states and the mechanism of their fracture were established. From the analysis of the obtained results, taking into account the known data, the following conclusions can be drawn:

1. Non-radial diffuse streaks and complex patterns of arrangement of diffuse satellites of type 1/2, 1/3, and 1/6 were revealed and systematically investigated in detail using the diffraction mode of high resolution TEM at a high accelerating voltage 300 kV.
2. The crystallographic mechanism of the martensite nucleation and rearrangements β_1_→β_1_′ and β_1_→γ_1_′ is proposed, based on the analysis of (i) the diffuse scattering patterns that occur in the pre-martensitic state and (ii) the internal defects of martensite substructure in these alloys.
3. It was revealed that SPD of metastable austenitic Cu-Al-Ni alloys via HPT of 6 GPa (with the number of revolutions from 1 to 10) leads to the creation of a deformation-induced UFG structure of martensite responsible for its high hardness and strength properties.
4. A subsequent annealing of moderate duration provides preservation of a UFG structure and strengthening of the alloys. The highest strength(σ_u_ up to 1400 MPa) and improved ductility-related (δ = 12–13%) properties were obtained in the UFG martensitic alloy Cu–14Al–3Ni either subjected to the HPT of 10 revs and short-term annealing at 1073 K for 10 s or subjected to the increased (to 423 K) HPT temperatures.
5. Annealing at temperatures below M_s_ initiates realization of the proeutectoid decomposition of martensite in the HPT-treated alloys with precipitation of an aluminum-enriched γ_2_ nano phase. The grain size, phase composition, and substructure of the martensite are preserved, but its depletion of aluminum by 1–2 at.% causes the stabilization of martensite and a noticeable increase in the critical temperatures of the TMT (by 70–160 K).
6. The annealing in the austenite state in the interval 570–840 K (above the temperature A_f_) leads to the combined reaction of primary nanorecrystallization accompanied by a heterogeneous preferably grain-boundary eutectoid (α + γ_2_) decomposition of the reverted β_1_ austenite into the homogeneous UFG (β_1_ + α + γ_2_) nano-triplex structure. Annealing at temperatures above T_ED_, which is close to 840 K, leads (due to a preferably heterogeneous precipitation of the γ_2_ nanophase) to the formation of a micro-duplex (β_1_ + γ_2_) structure. When cooled to RT, the residual β_1_ austenite experiences the TMT.
7. According to the data of fractographic investigations, the alloys in a UFG state are distinguished by their ductile–brittle character of fracture with a high degree of dispersion of separate dimples along high-angle boundaries of the ensembles of nano grains unified by close-in-value, small-angle misorientations.

## Figures and Tables

**Table 1:** Chemical spectroscopic analysis of the alloys Cu–14Al–3Ni and Cu–13.5Al–3.5Ni.

No.	Nominal Composition	Al, wt%	Ni, wt%	Fe, wt%	Cu
1	Cu-14Al-3Ni	13.95	3.02	-	83.03
2	Cu-13.5Al-3.5Ni	13.40	3.36	0.05	83.19

**Table 2:** Critical temperatures at the start (M_s_, A_s_) and end (M_f_, A_f_) of the TMT in the alloy Cu–14Al–3Ni after different treatments (see Figure 8a and curves 1, 2 in Figure 8b).

Treatment	M_s_, K;	M_f_, K	A_s_, K	A_f_, K	ΔT, K
Quenching from 1223 K (ρ(T))	250	230	265	280	33
Quenching from 1223 K (χ(T))	255	240	265	280	25
HPT of 10 revs (1)	320	300	400	440	110
HPT of 10 revs (2)	-	-	380	470	-

ΔT = 1/2{(A_s_ + A_f_) − (M_s_ + M_f_)}.

**Table 3:** Average size of grains of the Cu-14Al-3Ni alloy subjected to HPT of 10 revs and to different anneals.

Treatment of Alloy	Average Grain Size <d_G_>, nm
HPT, *n* = 10 revs	30
HPT + 373 K, 30 min	30
HPT + 473 K, 30 min	30
HPT + 573 K, 30 min	100
HPT + 673 K, 30 min	150
HPT + 773 K, 30 min	350
HPT + 873 K, 30 min	400
HPT + 1073 K, 10 s	3500

**Table 4:** Dependence of the microhardness of the Cu–14Al–3Ni alloy on the temperature of annealing for 30 min after quenching and HPT of *n* = 5 revs.

Temperature of Annealing, K	H_V_, GPa	
	Quen. 1223 K;	Quen. 1273 K;
-	4.70	4.22
	**Q + HPT, *n*=5**	**Q + HPT, *n* = 5**
-	5.10	4.60
473	5.65	5.25
573	5.85	5.55
673	5.70	5.40
773	5.10	4.90
873	4.40	4.00

**Table 5:** Dependence of the microhardness of the Cu-13.5Al-3.5Ni on the temperature of annealing for 30 min after quenching and HPT of *n* = 10 revs.

**T, K**	290	373	473	523	573	623	673	723	773	823	873
**H_V_, GPa**	5.0	5.5	5.13	5.33	5.58	5.9	6.1	5.93	5.55	5.03	4.02

**Table 6:** Results of tensile mechanical tests of the Cu-14Al-3Ni alloy after different deformation–temperature treatments.

Treatment	σ_M_, MPa	σ_u_, MPa	ɛ_M_, %	δ, %
Quenching from 1223 K	160	620	2	7
Quenching from 1273 K	60	400	2	11
HPT, 10 rev, (293 K)	-	820	-	4
HPT, 10 rev, (423 K)	-	1450	2	12
HPT 10 rev + 573 K, 30 min	120	450	2	6
HPT 10 rev + 773 K, 30 min	50	320	3	8
HPT 10 rev + 1073 K, 10 s	250	900	5	13

## Data Availability

All data included in this study are available upon request by contact with the corresponding author.
